# Study of the interactions of sneezing droplets with particulate matter in a polluted environment

**DOI:** 10.1063/5.0067517

**Published:** 2021-11-09

**Authors:** Prasenjit Dey, Sandip K. Saha, Sandip Sarkar

**Affiliations:** 1 Department of Mechanical Engineering, National Institute of Technology Goa, Ponda, Goa 403401, India; 2 Department of Mechanical Engineering, Indian Institute of Technology Bombay, Mumbai 400076, India; 3 Department of Mechanical Engineering, Jadavpur University, Kolkata 700032, India

## Abstract

We have performed a three-dimensional numerical simulation to determine the effect of local atmospheric pollution level on the spreading characteristics of the severe acute respiratory syndrome coronavirus 2 (SARS-CoV-2) virus through ejected droplets during sneezing and coughing in an open space. Utilizing a finite volume-based numerical method, we have performed computations for various ranges of droplet diameters and sneezing speeds. The interactions between the droplets and the suspended particles are considered by taking both hydrophobic and hydrophilic wettability characteristics into account. Our computational results show that the virus-containing droplets partially affect aerosols during the path of their transmission. With the progression of time, the droplet distribution shows an asymmetric pattern. The maximum dispersion of these droplets is found for higher sneezing velocities. The droplets with a diameter of 50 *μ*m travel a larger distance than the larger diameter droplets. We have found that an aerosol with hydrophilic wettability undergoes complete wetting by the disease-containing droplets and therefore is conducive to disease propagation. The droplet engagement duration with aerosol decreases with increase in the sneezing velocity. Our study recommends against using physical exercise centers in a closed environment such as gymnasium and indoor games during the COVID pandemic, especially in a polluted environment. The results from our work will help in deciding proper social distancing guidelines based on the local atmospheric pollution level. They may act as a precursor in controlling further spread of diseases during this unprecedented situation of the COVID pandemic.

## I. INTRODUCTION

The world is currently facing multiple waves of the COVID-19 pandemic with the new variants of coronavirus. It is a well-known fact that the severe acute respiratory syndrome coronavirus 2 (SARS-CoV-2) virus responsible for the COVID-19 pandemic spreads via respiratory droplets and enters the body through ingestions or inhalation of contaminated droplets. Therefore, the primary origin of this viral infection is coughing, sneezing, talking, and touching infected surfaces. A social distancing policy of 1.83 m (6 feet) is recommended for controlling airborne transmission based on the consideration of static ambient air; whereas, in reality, the atmosphere contains pollutants that influence respiratory droplet transport during its transmission. The pollutant levels at various local ambient air locations ranging from densely populated cities, to suburban, industrial cities, and villages can significantly influence the complexity of these social distancing norms. That, in turn, poses potential risks to the individuals exposed to the SARS-CoV-2 infection.

The larger droplets originate from saliva during coughing or sneezing, whereas the smaller droplets originate in the vocal cords and mucous coating of the lungs. Previous studies have shown that the respiratory droplets do not follow independent trajectories. Instead, they are carried forward among warm, moist gas of turbulent clouds. The situation becomes interesting when the droplets directly collide with the solid particles present in air. To understand this, Banitabaei and Amirfazlia^1^ conducted an experimental and numerical study on the collision of a moving droplet with spherical hydrophobic solid particles in mid-air. The authors employed coupled level set and Volume of Fluid (VOF) scheme to understand the effects of droplet-to-particle diameter ratio, liquid viscosity, and the surrounding gas on the impact outcomes and the lamella formation process. They concluded that larger droplet-to-particle diameter ratios increase the collision time; however, they insignificantly affect the impact scenario. Later, Arumuru *et al.*^2^ conducted experiments to visualize the effect of sneezing and to test the effectiveness of face masks, which was replicated using a jet with a Reynolds number of 30 000 released from a circular tube. The authors demonstrated the spreading of the infectious pathogen could be significantly affected by N95 masks in the forward direction.

Several numerical and analytical investigations are performed to understand the transport of droplets in indoor and outdoor conditions. A numerical study by Abuhegazy *et al.*^3^ reported the aerosol transport and surface deposition in a classroom with dimensions of $9\times 9\times 3{\;m}^{3}$ (height) to mitigate the spread of COVID-19 and predicted the effects of particle size, source location, presence of glass barriers/screens, opening windows. The Lagrangian approach was adopted to track discrete particles in a continuous phase. Since the distribution of aerosol in classrooms is non-uniform and strongly depends on the layout of the air-conditioning system, the opening of windows helped in the removal of particles from the room significantly. Dbouk and Drikakis^4^ (2020) employed the compressible multiphase mixture Reynolds-averaged Navier–Stokes (RANS) equations and the *k*−*ω* turbulence model to investigate the mechanism of airborne droplet spreading in different wind conditions. The authors considered the initial size distribution of saliva droplets in their numerical model to track these droplets in the Lagrangian framework. They found that the wind speed significantly affects the propagation of airborne droplets in an open environment. In their later work,^5^ the authors analyzed the dynamic behavior of the transmission of airborne droplets using the same model based on the coupled Eulerian–Lagrangian approach. The authors demonstrated that wearing masks can reduce the traveling distance of respiratory droplets. Recently, Agrawal and Bhardwaj^6^ analytically analyzed the entrainment of the surrounding air in a jet-like cough cloud arising from humans and found that the cough cloud takes 5–8 s before dissipating regardless of the absence or presence of masks. Verma *et al.*^7^ experimentally investigated the efficacy of face masks with exhalation valves and face shields in mitigating the spread of airborne infectious droplets. The authors noted that the use of high-quality cloth face masks and surgical masks is preferred to stop the spread of COVID-19. Busco *et al.*^8^ predicted the spread of droplets from human sneeze numerically, considering the coupled Eulerian–Lagrangian momentum equations. The Lagrangian method was employed to model the droplets arising from human sneeze. Moreover, the authors studied the effect of suspended particulates such as PM2.5 and PM10 on the sneezing dynamics by considering the momentum interaction between the Lagrangian phase of the particulate matter and the Eulerian phase of the continuum phase. The authors noted that increase in the particulates in air causes an increase in the spatial concentration of sneeze clouds. Tang and Min^9^ developed a theoretical model to investigate the transient water film evaporation on a spherical solid particle. Sen and Singh^10^ investigated the effect of various parameters such as wind speed, wind direction, ventilation near the door, and the extent of door opening on the spread of micro-sized droplets due to sneezing and coughing during human-to-human doorstep interactions. The authors pointed out that ventilation through windows near the door on a windy day reduces the degree of exposure to the airborne virus. The most recent research reported an alarming revelation on the dominance of SARS-CoV-2 through airborne transmission, where the possibility of infectious aerosols being carried away at various local atmospheric situations becomes of interest. Therefore, studying the possible consequences of airborne transmissions of the SARS-CoV-2 virus under different aerosol concentrations is crucial. The corresponding inter-droplet–particle collision mechanism on their transport characteristics, so far, has been ignored by the previous research studies. We believe such a study would bridge the gap in the literature and be expected to provide ready reference data to the public health community for immediate preventive action.

Khosronejad *et al.*^11^ studied the vorticity dynamics of human breathing using the Eulerian approach. The authors concluded that human saliva droplets can travel over 2.2 m from the origin of the sneezing source without a mask. However, the distance can be substantially reduced to 0.7 m by using non-medical grade face masks. Similarly, Das *et al.*^12^ evaluated the transmission of sneezed droplets with air flow and concluded that the lighter droplets (2.5 *μ*m of radius) can travel a larger distance with a wind flow of 0.1 m/s and can remain suspended in air for a longer time. Lee *et al.*^13^ evaluated the effectiveness of face mask microstructure and air flow on the dispersion of droplets arising due to sneezing. The droplets can travel a distance of 20–25 cm even when wearing face masks; hence, a distance of 20 cm can be considered safe. Chea *et al.*^14^ developed a numerical model using the multiphase turbulence model to predict the optimal physical distance depending on the indoor environmental wind conditions. The authors found that a distance of 5.8 m or more when sneezing is present in a gentle breeze at 4.5 m/s.

Liu *et al.*^15^ simulated the droplet distribution in a restaurant using large-eddy simulations (LESs) and suggested that improving the filtration effectiveness of an air-conditioning or ventilation system is very important to reduce the total infection risk depending on the ventilation and thermal conditions. Burgmann and Janoske^16^ using OpenFOAM conducted an investigation on the transmission of aerosols produced from an infected person in a classroom. The air purifier system can be used to reduce the airborne particles significantly in a classroom depending on the position of the infected person. Dbouk and Drikakis^17^ investigated the effectiveness of air purifiers in removing the airborne virus in a confined space and noted that air purifiers have minimal effect on airborne transmission; however, the virus removal effectiveness increases with the addition of inlet and outlet air ventilation. The same authors, Dbouk and Drikakis,^18^ examined the effect of climate on the virus transmission using three cities: New York, Paris, and Rio de Janeiro. The authors predicted two pandemic outbreaks per year depending on the temperature, relative humidity, and wind speed. Nazari *et al.*,^19^ using the RANS model in OpenFOAM, determined the safe pathway in an underground car parking area for better protection against viral transmission and highly recommended to wear a face mask in the parking area. A similar recommendation was made by Pendar and Páscoa^20^ while investigating the distribution of saliva droplets carrying the virus. Sen^21^ investigated cough droplet transmissions and their evaporation in an elevator. The author emphasized the use of proper ventilation, which causes the falling of droplets within a short distance from the source before infecting the other people in the elevator. Similarly, Zhang *et al.*^22^ highlighted the importance of opening doors and windows of confined spaces to reduce the transmission of aerosol carrying viruses. Shah *et al.*^23^ experimentally evaluated the effectiveness of masks and ventilation and suggested that high-capacity indoor ventilation can reduce aerosol mitigation. Wu *et al.*^24^ numerically analyzed the spreading of droplets from a sneeze in a cafeteria. It was found that further spreading of droplets occurs due to horizontal air flow from an air conditioner.

Mittal *et al.*^25^ developed a mathematical model to estimate the transmission of airborne droplets affecting the human respiratory system considering a single host. Their model was able to show the reduction in transmission of droplets by following physical distancing and wearing different kinds of masks. Later experimental work of Simha *et al.*^26^ measured the distance traveled by droplets from cough. The authors developed a universal non-dimensional velocity profile of the jet generated due to coughing by different subjects.

It can be noted from the literature review that several authors have extensively studied the transport of droplets in air. However, the effect of suspended particles in the air and their interactions with the infection-carrying droplets have not yet been reported in the published literature. It is very important to understand the movement of aerosols along with the sneezing droplets as the aerosols can carry droplets along with them depending on the wettability of the aerosols. This phenomenon can increase the suspension time of the droplet in the air, which further can enhance the infection risk in a highly polluted situation. Additional consequences may arise due to the local atmospheric velocity, eventually enhancing the droplet interaction time with the pollutant particles. The overall effect may culminate in maneuvering the disease transmission path and thus affecting the safety norms for social distancing. However, this kind of research is not explored in the available studies as per the authors' best knowledge. Accordingly, to fill this gap, we performed three-dimensional computations in the present research to understand the transport of such infected droplets and their interactions with aerosol particles in the local atmosphere. Under various conditions, we present different scenarios on the local spread of droplets and their residence times after being ejected from the source during a sneeze or cough.

## II. NUMERICAL MODELING

### A. Definition of the physical problem

**FIG. 1.**
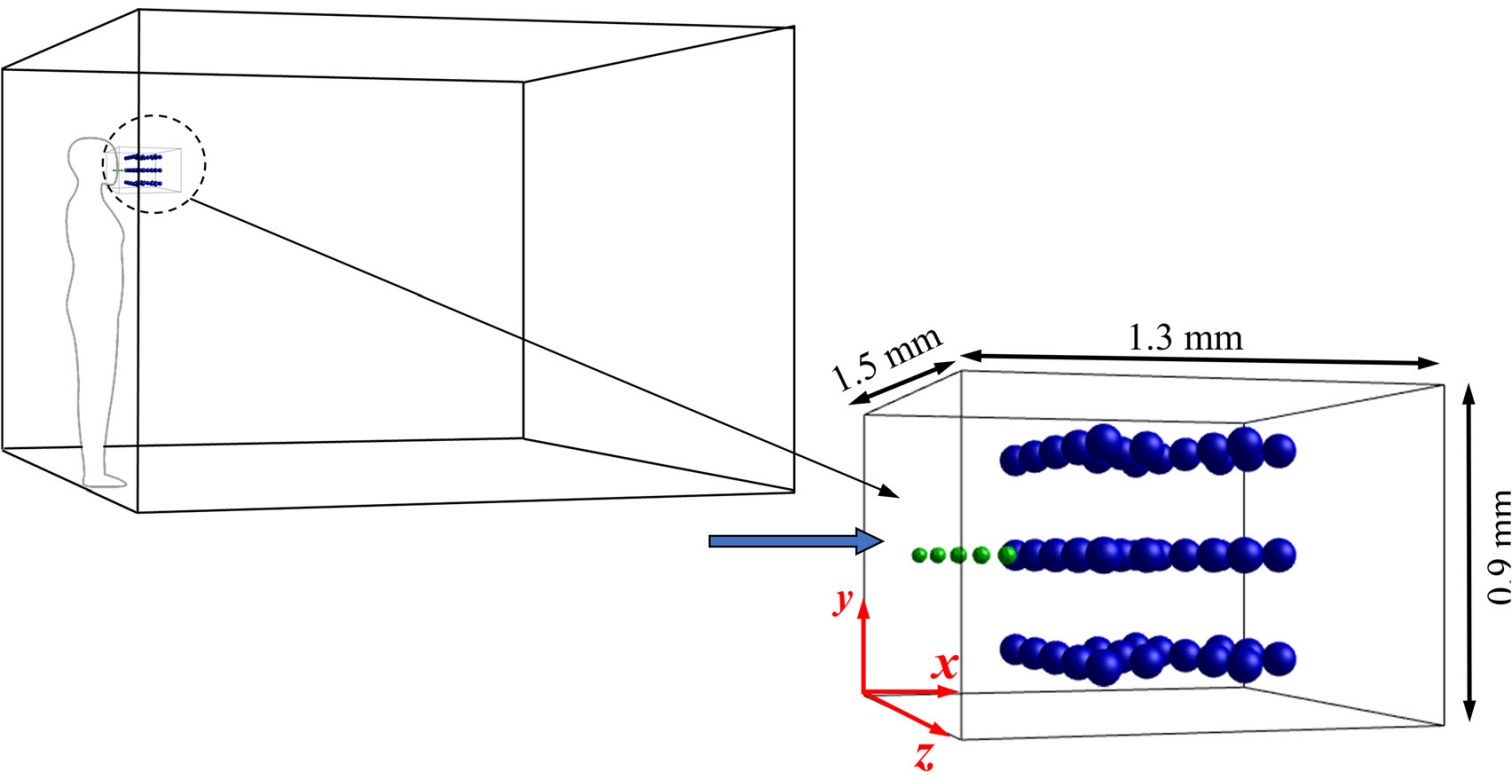
Schematic of the computational domain used in this research.

The actual condition of sneezing droplet propagation by the surrounding air flow is mimicked in the present study in a small volume, as shown in Fig. 1. A three-dimensional computational domain is considered. The size of the domain considered is given by width × height × length (*W × H × L*) *=* 150 *D_p_* × 90 *D_p_* × 130 *D_p_*, and *D_p_* is the diameter of the solid particles (pollutants). The effects of droplet diameter, solid particle (pollutant) diameter, and wettability of pollutants are investigated. At the initial condition, five sneezing droplets of diameter *D_d_* = 0.5 *D_p_*, 1 *D_p_*, and 1.5 *D_p_*, are placed near the mouth of a person (near the inlet). The diameters of the sneezing droplets are considered based on the smaller, medium, and larger types of droplets defined by Liu *et al.*^27^ The pollutants or the solid particles are considered a sphere of diameter *D_p_*, and 48 particles are considered in the present study. The position of each droplet and particle is shown in Fig. 1. The boundary conditions associated with the present numerical analysis are as follows:

(1) At the left wall of the computational domain, velocity inlet boundary condition is applied, where the air is allowed to enter the domain at a velocity of *U_in_*.
(2) The bottom, top, and right walls are assigned the pressure outlet boundary conditions so that the fluid and the particles can move out from the domain. This type of boundary condition relates to the actual condition where the fluid and the droplets are moving around the domain.
(3) On the side surfaces are allocated the symmetry type boundary conditions.

### B. Mathematical formulations

As shown in Fig. 1, the physics of the present research constitutes the interactions between the dispersed droplets and the solid particles suspended in the air. Accordingly, two different phases are initially considered inside the considered domain: (1) ambient air and (2) liquid water. Thus, a very well-known two-phase model, Volume of Fluid (VOF),^28^ is considered for numerical modeling. The VOF model can model two or more immiscible fluids by solving a single set of momentum equations and tracking the volume fraction of each of the fluids throughout the domain. The VOF formulation relies on the fact that two or more fluids (or phases) do not interpenetrate. In each control volume, the volume fractions (*α*) of all phases sum to unity, i.e.,

$$\alpha_{a}+\alpha_{w}=1,$$ (1)

where *α_a_* is the volume fraction of air and *α_w_* is the volume fraction of liquid water.

The fields for all variables and properties are shared by the phases and represent volume-averaged values, as long as the volume fraction of each of the phases is known at each location. Thus, the variables and properties in any given cell are either purely representative of one of the phases, or representative of a mixture of the phases, depending upon the volume fraction values and can be expressed as follows:

$${\emptyset =\alpha }_{a}\emptyset_{a}+\alpha_{w}\emptyset_{w}.$$ (2)

Here, ∅ can be *ρ* and *μ*. The value of ∅ in Eq. (2) in a cell ranges from 0 to 1. ∅ = 1, if the cell is occupied by the primary phase, whereas ∅ = 0 if the cell is filled with the secondary phase. At the interface, the value of ∅ is between 0 and 1. Here the volume fraction $\left(\emptyset \right)$ is a scalar function and can be computed from a separate transport equation in which the transport takes place in a purely convective manner as follows:

$$\frac{\partial \emptyset }{\partial t}+\nabla .\left(\vec{u}\emptyset \right)=0.$$ (3)

The main assumptions used for the simulation of particle transport include the following: (i) the airflow is isothermal and (ii) the evaporation of particles is neglected for simplification. The flow is assumed to be incompressible, Newtonian, unsteady, and turbulent. Based on these assumptions, the governing equations of the flow are as follows:

Continuity equation:

$$\nabla .\left(\vec{u}\right)=0.$$ (4)

Momentum equation:

$$\frac{\partial \vec{u}}{\partial t}+\nabla .\left(\vec{u}\vec{u}\right)=-\frac{1}{\rho }\nabla p+\frac{1}{\rho }\nabla .\left[\left(\mu +\mu_{T}\right)\left(\nabla \vec{u}+\nabla {\vec{u}}^{T}\right)\right]+{\vec{g}+\frac{1}{\rho }\vec{S}}_{m},$$ (5)

where *μ* is the molecular viscosity; *μ_T_* is the turbulent viscosity; *g* is the gravitational force; and *S_m_* represents the volumetric force due to surface tension, which is evaluated using the continuum-surface-force (CSF) model.^29^ The vapor–liquid interface is treated as a transition region of finite thickness, and the effect of surface tension is modeled as a volume force. The surface normal is computed as a gradient of the volume fraction scalar; then, the surface curvature *k*_c_ is obtained as follows:

$$k_{c}=\nabla .\hat{n},$$ (6)

where $\hat{n}$ is a unit vector normal to the interface.

The volumetric force source term in Eq. (5) is calculated as follows:

$${\vec{S}}_{m}=\sigma k_{c}\nabla \emptyset \frac{\rho }{\frac{1}{2}\left(\rho_{a}+\rho_{w}\right)}.$$ (7)

The wall adhesion angle is also considered along with the CSF model for investigating the effect of pollutant wettability on the sneezing droplets. In this model, the contact angle that the fluid is assumed to make with the solid surface is used to adjust the surface normal in cells near the surface. If *θ* is the contact angle at the wall, then the surface normal at the adjacent cell next to the wall is calculated as

$$\hat{n}={\hat{n}}_{c}c\text{os }\theta +{\hat{t}}_{c}s\text{in }\theta ,$$ (8)

where ${\hat{n}}_{c}$ and ${\hat{t}}_{c}$ are the unit vectors normal and tangential to the surface, respectively. Combining this contact angle with the usually calculated surface normal one cell away from the wall determines the local curvature of the surface. This curvature is used to adjust the body force term in the surface tension calculation.

#### 1. Turbulence model

The turbulence is modeled using the standard *k*–*ε* model with the standard wall function. The standard *k*–*ε* model is a semiempirical model based on model transport equations for the turbulence kinetic energy *k* and its dissipation rate *ε*. The model transport equation for *k* is derived from the exact equation, while the model transport equation for *ε* is obtained using physical reasoning and bears little resemblance to its mathematically exact counterpart.^30^ The turbulence kinetic energy, *k*, and its rate of dissipation, *ε*, are obtained from the following transport equations:

$$\frac{\partial }{\partial t}\left(k\right)+\frac{\partial }{\partial x_{i}}\left(k\vec{u}\right)=\frac{\partial }{\partial x_{j}}\left[\frac{1}{\rho }\left(\mu +\frac{\mu_{T}}{\sigma_{k}}\right)\frac{\partial k}{\partial x_{j}}\right]+{\frac{1}{\rho }G}_{k}-\varepsilon -\frac{1}{\rho }Y_{M},$$ (9)

$$\frac{\partial }{\partial t}\left(\varepsilon \right)+\frac{\partial }{\partial x_{i}}\left(\varepsilon \vec{u}\right)=\frac{\partial }{\partial x_{j}}\left[\frac{1}{\rho }\left(\mu +\frac{\mu_{T}}{\sigma_{\varepsilon }}\right)\frac{\partial \varepsilon }{\partial x_{j}}\right]+C_{1\varepsilon }\frac{1}{\rho }\frac{\varepsilon }{k}G_{k}-C_{2s}\frac{\varepsilon^{2}}{k},$$ (10)

where *G_k_* represents the production of turbulence kinetic energy; *Y_M_* represents the effect of the fluctuating dilatation on the overall dissipation rate; *σ_k_* and *σ_ε_* represent the turbulent Prandtl numbers for *k* and *ε*, respectively; and *C*_1__*ε*_ and *C*_2__*s*_ are empirical constants. The turbulent (or eddy) viscosity, $\mu_{T}$, is then computed by combining *k* and *ε* as follows:

$$\mu_{T}=\rho C_{\mu }\frac{k^{2}}{\varepsilon }.$$ (11)

#### 2. Modeling of pollutants (solid particles)

As the solid particles suspended in the air need to be allowed to move freely by the fluid, a dynamic mesh model is considered to model these particles' flow. The particles are designated as a rigid body with six degrees of freedom (DOFs), i.e., three translational degrees of freedom (DOFs) of the mass center and three independent rotational degrees of freedom of the three relative axes. The six-DOF motion of the dynamic mesh model is captured using the six DOF solvers in ANSYS Fluent.^30^ The change in mesh due to the movement of particles inside the domain is updated in the deformable mesh zone over time using a coupling of spring-based smoothing and local cell remeshing method.^30^ The considered computational domain is divided into two parts for the dynamic mesh model: the rigid body (solid particles) and the deformable mesh zone, where meshes are deforming with time and are constituted by tetrahedral types.

### C. Numerical solution methodology

The numerical analysis is carried out in a commercial finite volume method-based solver ANSYS Fluent 2020R2.^30^ The flow is assumed to be turbulent, and for modeling the same, the standard *k*–*ε* model with the standard wall function is adopted in this study. As the numerical model considers air (ambient fluid) and respiratory droplets (water), the two-phase model, Volume of Fluid (VOF), is considered. The Pressure-Implicit with Splitting of Operators (PISO) algorithm, which is an extension of the Semi-Implicit Method for Pressure Linked Equations (SIMPLE) algorithm, is used for pressure–velocity coupling.^31,32^ The PISO algorithm is a procedure for calculating the pressure–velocity coupling for the Navier–Stokes equations, which was developed initially for the non-iterative computation of unsteady compressible flows. However, this algorithm is successfully adapted to various unsteady incompressible single-phase and multiphase flow problems.^33–37^ The second-order upwind discretization scheme is considered for spatial discretization of the momentum equations and the transport equations of turbulence kinetic energy, *k*, and its rate of dissipation, ε. An adaptive time step of 10^−7^ to 10^−9^ is considered in the present study by fulfilling the Courant–Friedrichs–Lewy (CFL) condition, CFL ≤0.5. All simulations are stopped at 5 ms or stopped earlier when the respiratory droplets have escaped from the considered numerical domain. Numerical simulations are performed on a workstation (Intel^®^Xeon^®^ Processor E5–2683 v4 with dual processors, 64 cores, and 256 GB RAM). The simulations took approximately three to four weeks to complete one simulation with a 5 ms flow time duration.

### D. Mesh details and mesh independence test

**FIG. 2.**
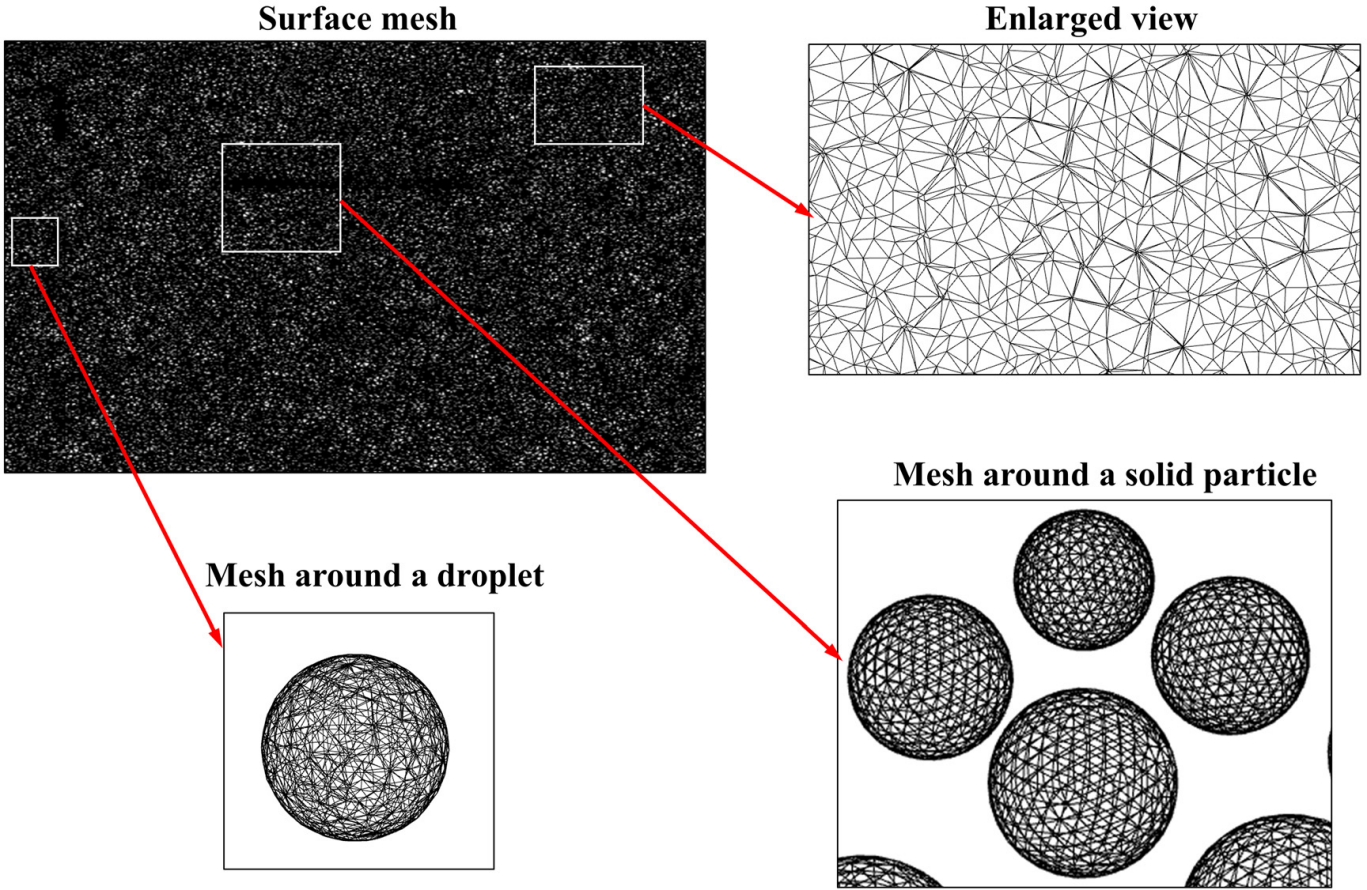
Meshing adopted in the present numerical domain.

An unstructured, tetrahedral mesh is used, as shown in Fig. 2. The present mesh is generated using ANSYS Meshing 2020R2.^30^ The mesh consists of 1.2 × 10^7^ mesh cells (a detailed mesh convergence study is discussed below) with a minimum cell size of 0.015*D_p_* to accommodate at least 3000 mesh cells in each droplet, and the corresponding image of mesh around a droplet is also shown in Fig. 2. Each solid particle consists of around 5000 mesh cells and is depicted in Fig. 2. To determine the proper mesh size for numerical analysis, a grid independence test is carried out using three mesh sizes: 6 × 10^6^, 1.2 × 10^7^, and 2.4 × 10^7^. Both the coarse and fine meshes yield a similar result.

### E. Model validation

**FIG. 3.**
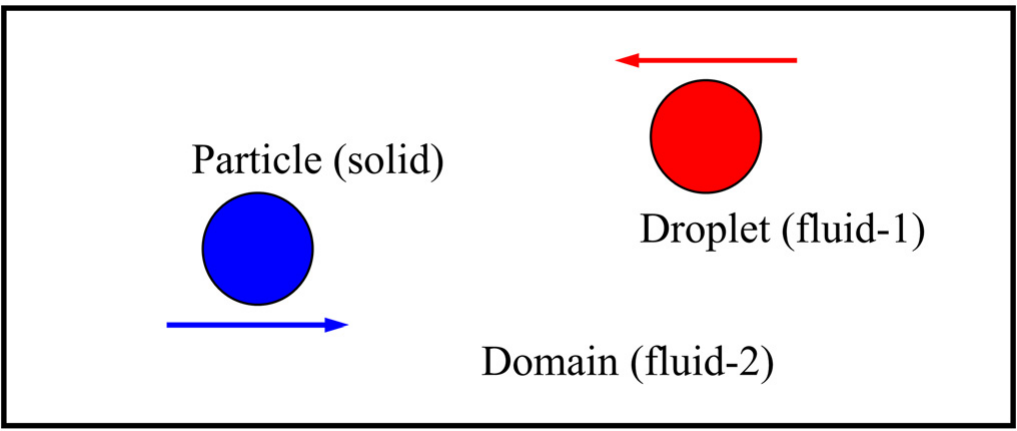
Schematic diagram of the investigated domain^38^ used for validation.

**FIG. 4.**
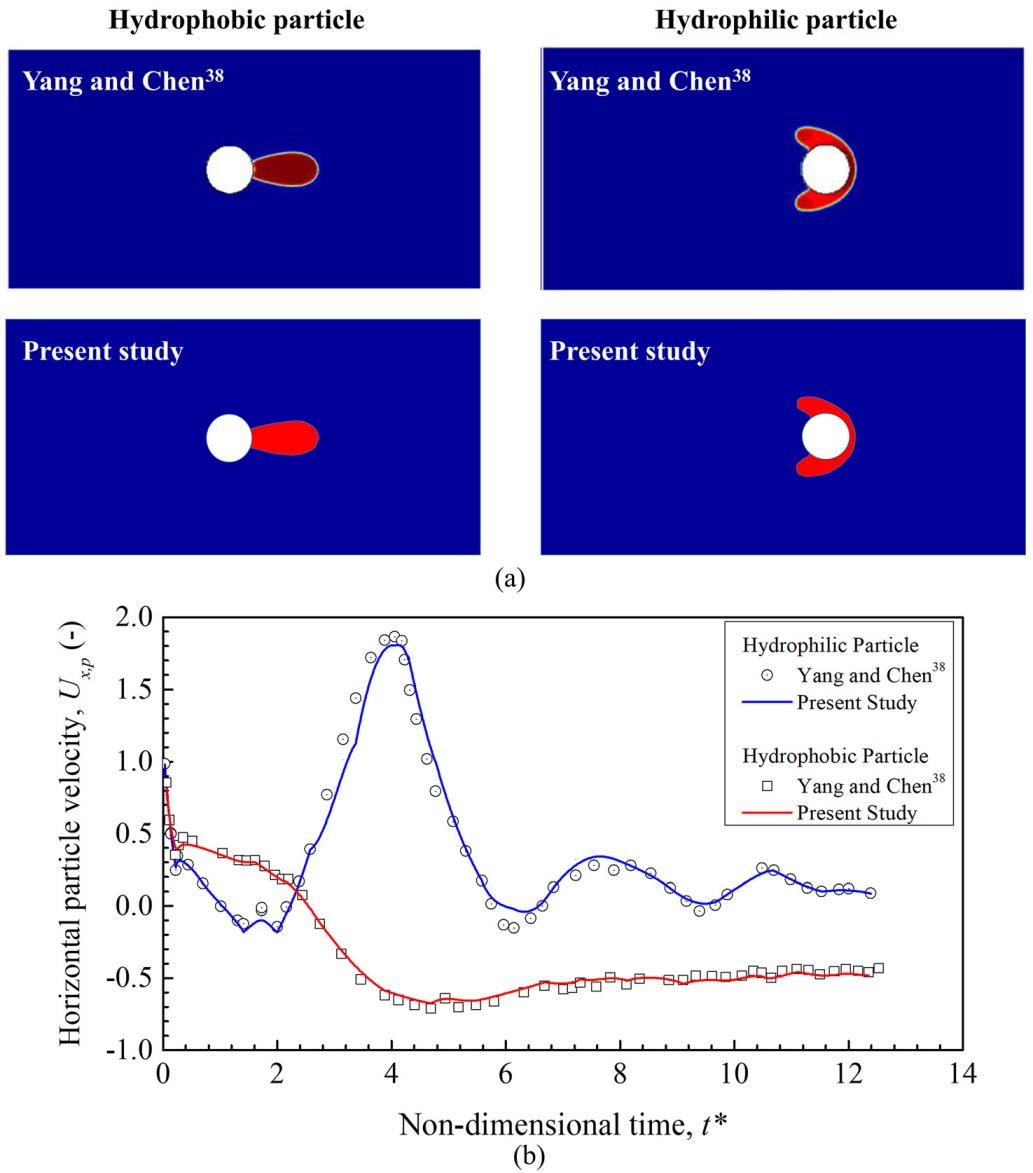
(a) Interaction of moving solid particle and liquid droplet at a non-dimensional time, 3.75, and (b) time-dependent velocity of the moving solid particle for hydrophobic and hydrophilic wetting characteristics.

In order to confirm the correctness of a numerical model, a comparative evaluation between the present numerical model with the published available model needs to be performed. As there are no data available for the movement of respiratory droplets in an open environment with moving solid particles (pollutants) of different wetting characteristics, a similar study of the interaction between a freely moving solid particle and a freely moving liquid droplet analyzed by Yang and Chen^38^ is considered for verifying the accurateness of the present numerical model. They investigated the interactions between a freely moving solid particle with different wettability and a freely moving liquid droplet in a different fluid domain, as shown in Fig. 3. Their model can be related to the present numerical model as the present model deals with a two-phase flow (air and water droplet), with moving pollutants (solid particles). However, the model is validated for a two-dimensional case, and the exact model is extended to the three-dimensional case for this study. Both the qualitative and quantitative data are considered for the validation of the present model. The interaction of a moving droplet with a moving solid particle for two different wetting characteristics, hydrophobic and hydrophilic, is shown in Fig. 4(a) at a non-dimensional time, 3.75. It can be observed that the droplet interaction with the solid particle captured in the present model is similar to the behavior of the droplet that Yang and Chen^38^ predicted. In addition, the time-dependent velocity of moving solid particles is also compared for two wetting parameters, hydrophobicity and hydrophilicity, and is shown in Fig. 4(b). It can also be noticed that the behavior of solid particle movement is captured well by the present model for both the wetting parameters with the data that are taken from the study of Yang and Chen.^38^ These well-predicted qualitative and quantitative data show the ability of the present model for analyzing the behavior of respiratory droplets in an open environment with pollutants.

## III. RESULTS AND DISCUSSION

One of the critical mechanisms in the spread of any virus, such as the SARS-CoV-2, is sneezing by an infected person in fresh air. The respiratory droplets transmit through the air and affect the nearby persons in contact with the droplets. The zone of influence depends on the path traveled by the droplets, which is further extended by the interactions of the droplets with the solid particles present in the air. The surface wettability of the particles determines the stages of wetting conditions with the droplets, thereby affecting the safety norms for social distancing. Accordingly, in the present research, we have performed three-dimensional computations of the droplet trajectories, focusing on their interactions with the suspended particles in the air. With the aim of exploring such consequences, the results are generated for the following range of conditions: the inlet velocity range is assumed as $5\leq U_{in}\leq 15$ and the wettability is varied in the range of 60°≤θ≤120°. The range of droplet diameter is considered as $50\;\mu m\leq D_{d}\leq 150\;\mu m$, and the diameter of the suspended particles in the air is considered as, *D_p_* = 100 μm. Multiphase three-dimensional simulations are performed for about 5 ms, and results are obtained in terms of droplet dynamics and their characteristics under various inflow conditions.

**FIG. 5.**
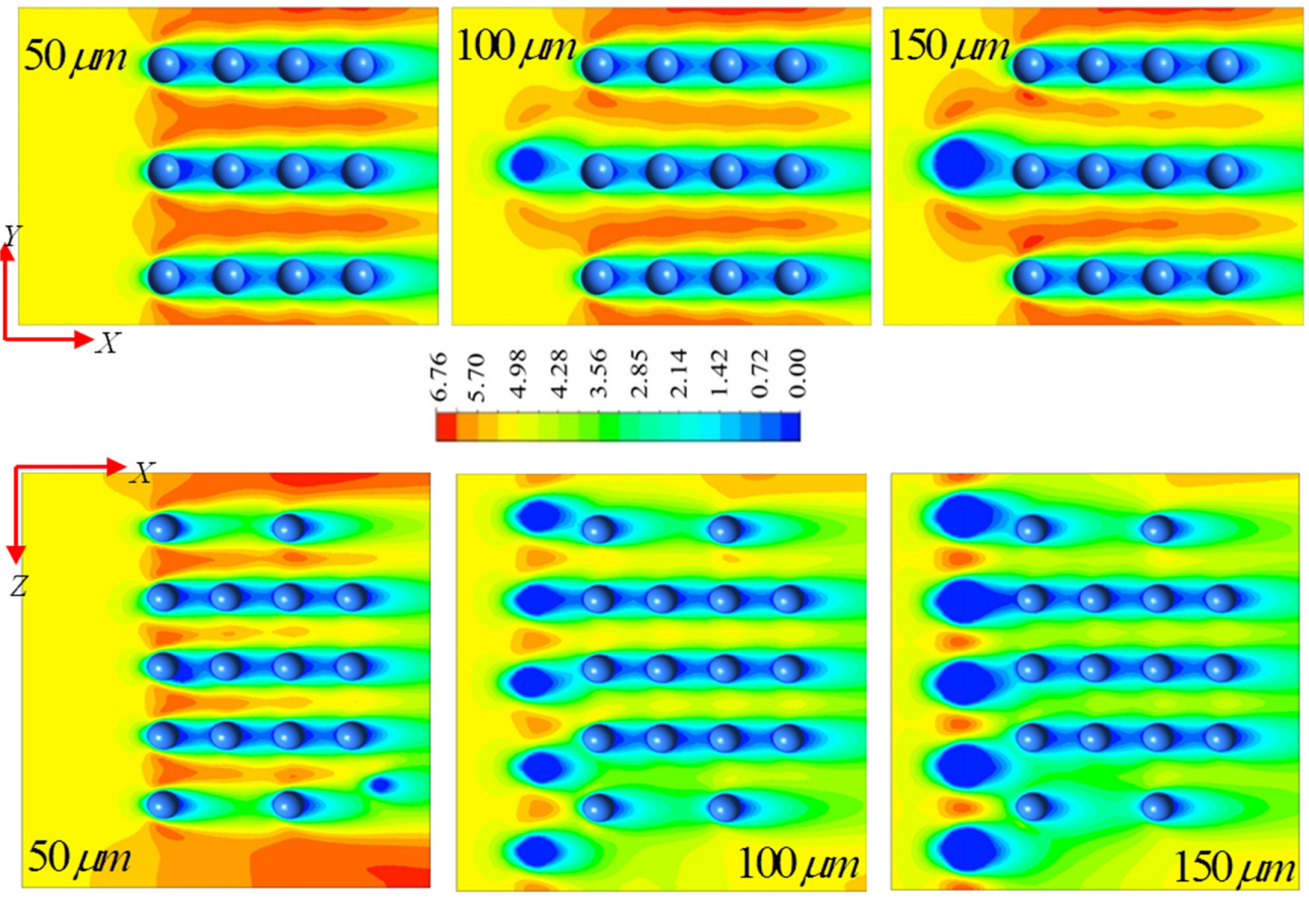
Normalized velocity $\left(u/u_{\text{max}}\right)$ contours at various droplet diameters in the *X–Y* and *X–Z* planes for $U_{in}=5\;m/s$ and θ=120°. The advection of respiratory droplets and their interactions with the suspended air particles are visible.

We obtain our results by first analyzing how the suspended particles in the atmosphere in the form of pollutants behave in the presence of respiratory droplets of various diameters. Figure 5 shows the normalized velocity $\left(u/u_{\text{max}}\right)$ contours at different droplet diameters in the *X–Y* and *X–Z* planes, respectively. The *X–Y* and *X–Z* planes are located midway along the *Z* and *Y* axes, respectively. We show the results for a representative value of $U_{in}=5\;m/s$ and θ=120°. The traveling respiratory virus droplet interact with the suspended particles in the air. The level of atmospheric pollution plays a crucial role in determining the degree of interaction. From the velocity contours in Fig. 3, it is clear that there is a rigorous interaction of the droplets with the suspended aerosols during their advection from the source. The airflow causes a change in the shape of the droplets. We see that the droplets, which are initially circular in shape during sneezing, suffer deformation and yield an elliptical contour. The streaming air velocity is displaced by the droplets, and as a result, there is a formation of a low-velocity region in the downstream direction. Accordingly, the aerosols occupying the downstream region also experience consequences of the low-velocity region around it. Nevertheless, there is a formation of a thin band of a high-velocity region between two aerosol particle groups. The effect is more prominent for the higher value of the droplet diameter of 150 μm, where it is seen that the zone of influence of the low-velocity region around the aerosol groups is the thickest. This is a significant result since the zone of influence also indicates the effective transmission region of the SARS-CoV-2 virus with the aerosols, which, in turn, eventually increases the social distancing limit. In this context, it may be mentioned that the respiratory droplets expelled by humans when coughing, talking loudly, or sneezing have an effective size ranging from 5 to 2000 μm. The droplet dispersion cloud is a function of the rigorousness of each action made by humans. The range of droplet sizes during talking of an average human being varies from 25 to 50 μm with 600 droplets expelled, whereas, during sneezing, this number increases up to 800. The size of the maximum number of droplets in sneezing, talking, or coughing may fall in the between 25  and 50 μm; however, larger droplets are relatively fewer in number. Although, in the expelled volume of the air, we note that the volume fraction of the droplets is significantly lesser.

**FIG. 6.**
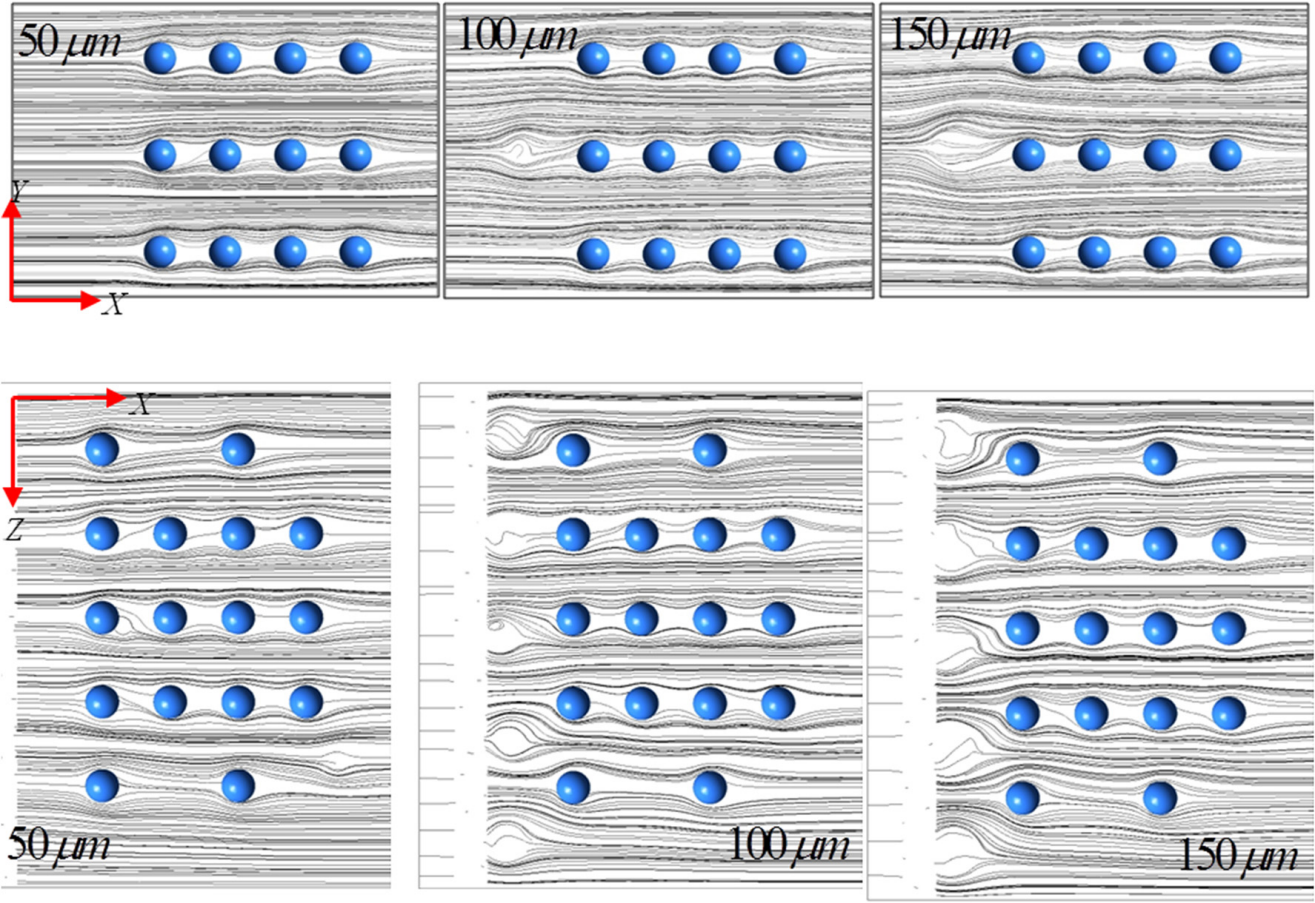
Streamline contours for various droplet diameters in the *X–Y* and *X–Z* planes, respectively. The streamlines are shown for a representative value of $U_{in}=5\;m/s$ and θ=120°.

Figure 6 shows the corresponding streamline contours for various droplet diameters in the *X–Y* and *X–Z* planes, respectively. The streamlines are shown for a representative value of *U_in_* = 5 m/s and θ=120°. The streamlines show the formation of saddle points in the downstream wake of the droplet for increasing diameters. The presence of aerosol particles downstream of the droplets causes further deflection of the streamlines and they eventually emerge as a surface streamline observed in the wake past a sphere. We do not observe any presence of the recirculation region in the back stagnation regions of the aerosol particles. Owing to the micrometer-sized aerosol particles, the streamlines are intercepted and form a void space between the evenly spaced particles. With increase in the droplet diameter, the streamlines are found to penetrate more in the void space between the aerosol particles. From these observations, it may be concluded that the virus-containing droplets partially affect the aerosols during their transmission; however, for a densely polluted area, there could be complete wetting of the particles depending on their sizes.

**FIG. 7.**
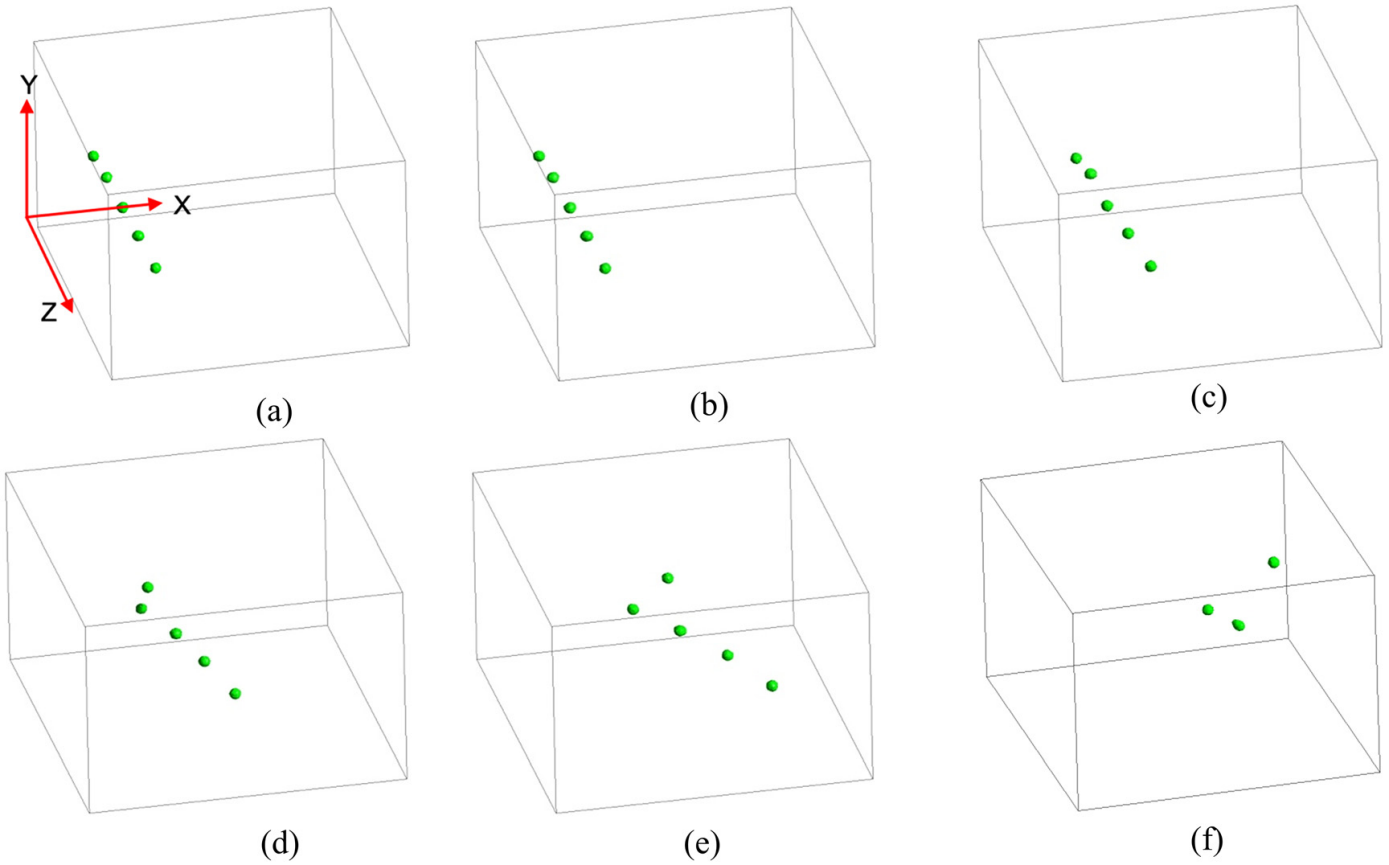
Transient distribution of droplet location at (a) *t* = 0 ms, (b) *t* = 0.5 ms, (c) *t* = 1 ms, (d) *t* = 1.5 ms, (e) *t* = 2 ms, and (f) *t* = 3 ms.

The temporal variation in droplet position for *U_in_* = 15 m/s, *θ* = 120°, *D_d_* = 50 *μ*m, *D_p_* = 2*D_d_* is shown in Fig. 7. In this figure, the aerosol position is not shown. The interaction mechanism between the droplets and the aerosols is discussed later. The initial position of the droplet at the time of sneezing is depicted in Fig. 7(a). At *t* = 0.5 ms, the droplets remain at the same position and these are not influenced by the air velocity due to sneezing. At *t* = 1 ms, the droplets are observed to have moved along the air flow direction. As time progresses, the droplet positions are significantly affected by the air flow. At *t* = 1.5 and 2 ms, the droplets are seen to start dispersing in the flow according to the flow distribution. The droplet distribution becomes asymmetric as time progresses. Apparently, droplets travel faster in the direction where there is bias in the streaming fluid. Again, the lateral distance within the droplets increases with advancement in time. Interestingly, we can observe that a certain group of droplets always precedes the rest. As a result, the overall distribution follows a triangular pattern. With progress in time, the distance among the droplets residing at the vertex increases to such an extent so that the overall coherence in maintaining the gross droplet distribution is lost, which is discernible at *t* = 3 ms. Again, the bias of the stream fluid velocity perhaps causes such consequences, which can be further observed from the streamline plots shown in Fig. 6. From the perspective of the spread of COVID-19 carrying droplet clouds, one may experience a higher degree of virus load from an infected person maintaining a higher distance than the existing social distancing norm of 1.83 m.

**FIG. 8.**
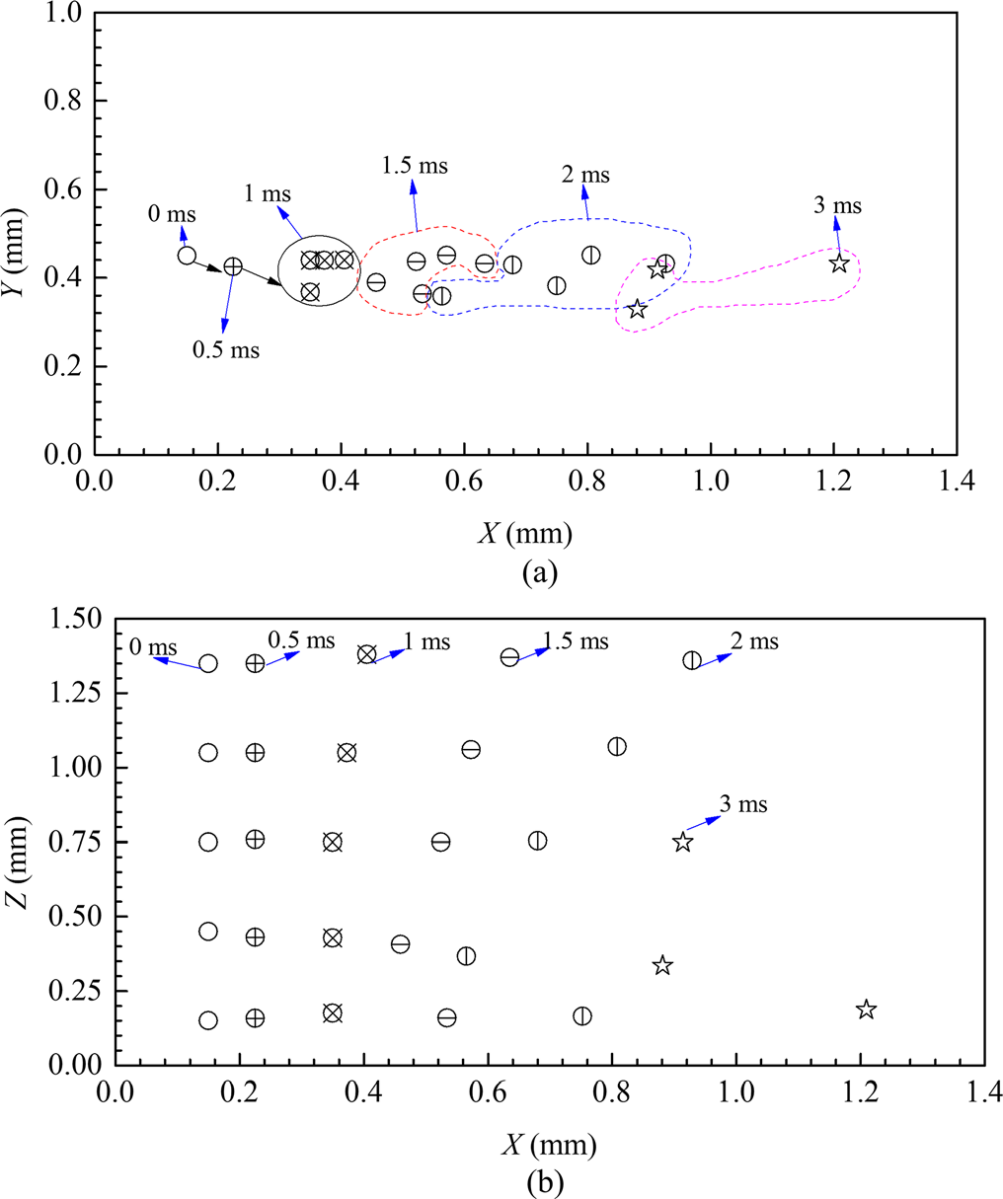
Distribution of particles at different times in the (a) X–Y and (b) X–Z planes.

Figures 8(a) and 8(b) show the variation in droplet position with time in the X–Y plane and the X–Z plane, respectively. Initially, the droplets are concentrated at one point at the source. Once the sneezing occurs, the particles start to disperse in the flow. It can be observed that the lateral dispersion of these particles is marginal with the progress of time. Accordingly, we can identify three droplet cluster groups based on time, as shown in the figure. In the first cluster group at *t* = 1 ms, the droplets are close to each other and travel as a cluster of particles in fractal groups. In the second group at *t* = 1.5 ms, dispersion of droplets occurs, causing the lateral distance between two droplet centers to increase. In the third group, at *t* = 2 ms, we observe that the droplets are more spread in the cloud. Subsequently, the spread of droplets with time may be observed, culminating in droplet redistribution in a localized space. However, the dispersion of particles is more in the X–Z plane, as shown in Fig. 6(b), compared to the X–Y plane. From Fig. 6(b), we can observe that starting from *t* = 0, there is an abrupt droplet distribution along the X–Z plane as time progresses. With increase in time, the droplets redistribute themselves and take the shape of a paraboloid from *t* = 1.5 ms. This also corroborates our earlier observation from Fig. 7. Interestingly, it can be noted that the gravitational effect causes the droplet to slightly fall from *t* = 1.5 ms, which could be the reason for asymmetricity in the spread of droplets.

**FIG. 9.**
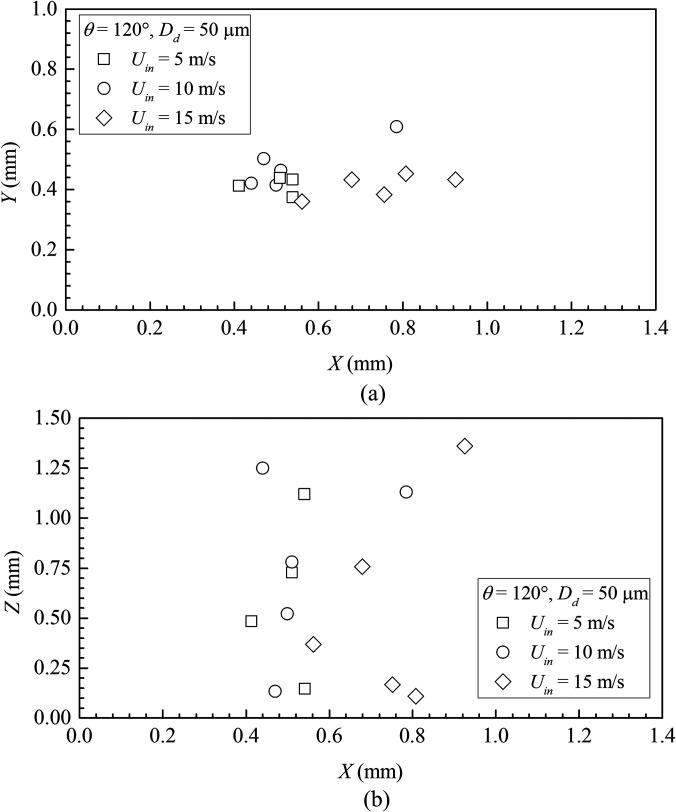
Droplet distribution for different sneezing velocities in the (a) X–Y and (b) X–Z planes.

We now proceed to understand how the sneezing velocity affects the droplet distribution from an infected person. Accordingly, in Fig. 9, we plot the effect of sneezing velocity on the distribution of droplets in the X–Y and X–Z planes for *U_in_* = 5, 10, and 15 m/s, θ = 120°, *D_d_* = 50 *μ*m, *D_p_* = 2*D_d_*, *t* = 2 ms. We observe many interesting consequences from the droplet distribution for varying sneezing velocities. In general, it is found that the droplets move faster with increase in the sneezing velocity. On the X–Y plane in Fig. 9(a), we observe that the droplets injected with a sneezing velocity of 15 m/s travel a much longer distance than the droplets with lower sneezing velocity. Interestingly, the effect of triggering the sneezing velocity does not have any consequences on the lateral dispersion of these droplets. However, droplets generated with the sneezing velocity of 10 m/s show a slightly higher dispersion than the rest of the sneezing velocities. This could probably be due to the effect of intra-droplet collision and spreading, which is also a function of the surface tension and the capillary pressure inside the droplets. Again, from Fig. 9(b), we observe the maximum dispersion of droplets along the X–Z plane. Droplets with the highest sneezing velocity show maximum dispersion compared to the other velocities considered in this study. The gravitational effects on the droplet fall are evident and do not depend on the sneezing velocity. Again, we can conclude that the safety norms for social distancing may alter if a person sneezes with a higher velocity. Such consequences are very much applicable to persons involved in regular sports activities. In contrast, a face mask significantly decreases the momentum of these particles, reducing further spread of droplets from the infected person.

**FIG. 10.**
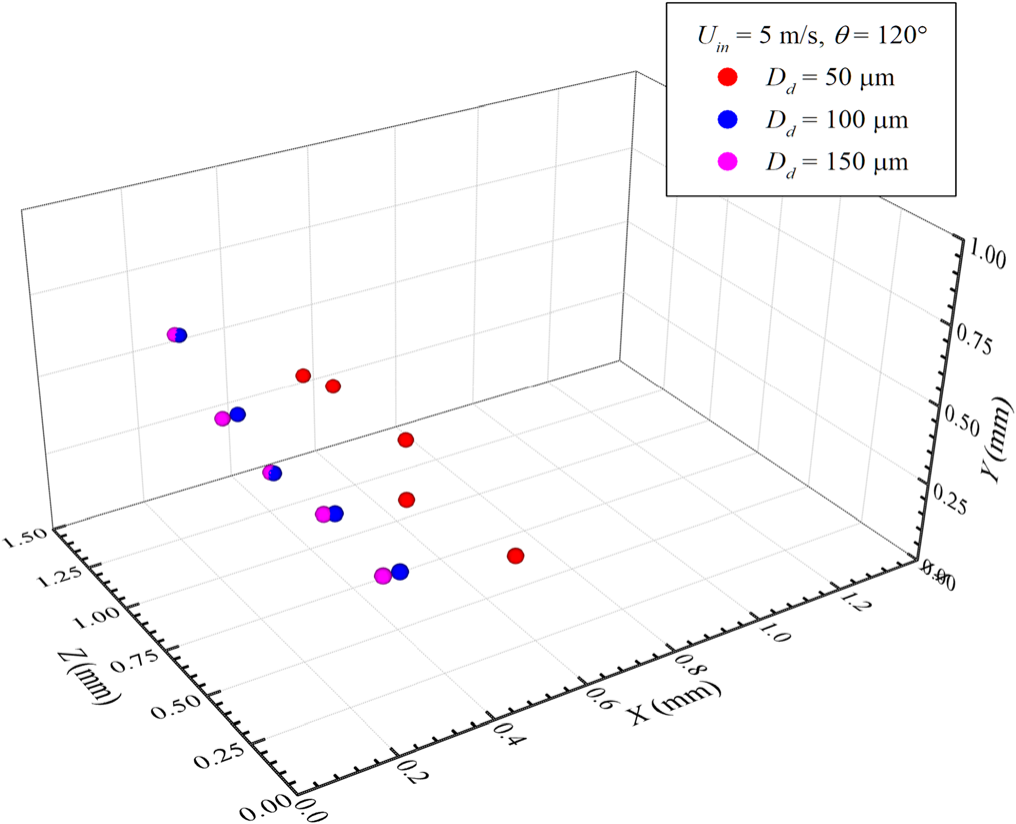
Effect of droplet diameter on its position.

It is known that during sneezing, a person does not sneeze equal-sized droplets. In reality, the droplets follow a specific size distribution. Such droplet size distributions do not follow a particular trend, rather vary from person to person. Therefore, it is not possible to accurately predict the droplet distribution for analysis. To understand the effect of droplet size distribution on the disease spreading characteristics, we have invoked an approximate analysis by considering three droplet sizes: 50, 100, and 150 *μ*m. Figure 10 shows the distribution of droplets in a three-dimensional space for a representative sneezing velocity of *U_in_* = 5 m/s and *θ* = 120° at *t* = 2 ms. We can see that the droplet having a diameter of 50 *μ*m covers a larger distance than the larger diameter droplets due to their higher inertia and less effect of drag force. It is known that the Strokes drag acting on a particle of diameter *D_d_* can be expressed as $D=3\pi D_{d}\mu u$. The drag force for *D_d_* = 50, 100, 150 *μ*m is found as 0.042, 0.084, and 0.126 *μ*N, respectively. Accordingly, we can see that the particle with a higher diameter experiences a larger drag force for a fixed inlet velocity and fluid viscosity, causing the particle to travel smaller distances. In this context, it may be mentioned here that the droplet range during human exhalation varies within 0.01–1000 *μ*m, which is classified into groups by the medical community for infectious diseases. The first group contains the respiratory droplets of diameter varying in the range of 5–10 *μ*m, whereas the second group contains droplets of 5 *μ*m diameter known as aerosols. The aerosols in general remain airborne for a longer time. In contrast, droplets fall quickly to the ground nearer to their source. In practice, the cutoff between the aerosols and the droplets is arbitrary.^39^

It is very important to understand the movement of aerosols along with the sneezing droplets as the number of aerosols varies throughout the year.^40^ It has been observed that the mass concentrations of PM10 and PM2.5 vary in the range of 20–500 *μ*g/m^3^ from monsoon to winter in Delhi, India,^40^ and the value can increase to 1200 *μ*g/m^3^. Hence, the interactions of sneezing droplets with the aerosols increase during high mass concentrations of PM10, and PM2.5, which need to be explored to understand the behavior of these droplets in such a highly polluted situation. In the present study, the movement of aerosols is also considered by implementing a dynamic mesh model, as discussed earlier. The mass of the aerosols is considered by satisfying the mass concentrations of 500 *μ*g/m^3^. The change in position of the aerosols is found negligible within the considered simulation time due to limitations in the computational power.

**FIG. 11.**
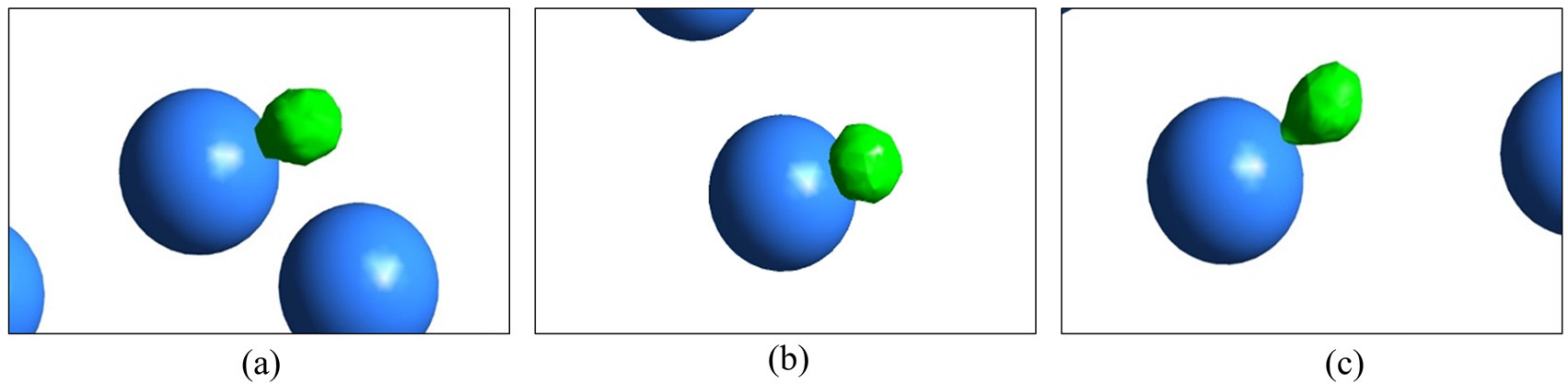
Droplet–particle interaction with time: (a) 1.8 ms, (b) 1.9 ms, and (c) 2 ms for *U_in_* = 10 m/s, *θ* = 120°, *D_d_* = 50 *μ*m, *D_p_* = 2*D_d_*.

We observe the different characteristics of droplet–particle interactions in aerosol in Fig. 11. As can be found from the figure, the droplet starts interacting with the particle at *t* = 1.8 ms after the droplet generation. Thereafter, the droplet spreads over the particle surface at *t* = 1.9 ms. Interestingly, at *t* = 2 ms, we found that the droplet starts to detach from the particle surface with a receding contact angle. Therefore, the attachment and detachment process of the droplet undergoes continuous-time marching steps. Notably, between *t* = 1.9 and 2 ms, the detachment process initiates. However, we could not capture the time point in the present research due to limitations in computational resources. From this analysis, we can perhaps conclude that it may not always be necessary that the aerosol would carry the infected droplets. Therefore, the infectious cloud may propagate an additional distance depending on the local pollution level and the constituent aerosol particles with their wettability characteristics. Such a consequence may affect the social distancing norms of a highly polluted, moderately polluted, and less polluted environment. To the best of our knowledge, this is the first time that such a claim on infection propagation through air is being reported.

**FIG. 12.**
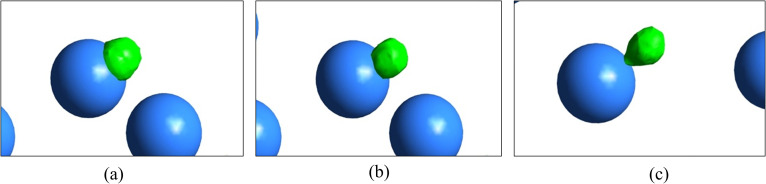
Effect of contact angle on the droplet–particle interaction for (a) 60°, (b) 90°, (c) 120° for *U_in_* = 10 m/s, *t* = 2 ms, *D_d_* = 50 *μ*m, *D_p_* = 2*D_d_*.

Again, the impact of contact angle on the spreading characteristics of the droplet is very much palpable from Fig. 12. It is observed that for *θ* = 60°, there is a complete spreading of the droplet, thus resembling hydrophilic characteristics of the particle surface. Therefore, aerosol having hydrophilic wettability characteristics is conducive to disease propagation. In reality, the atmospheric air pollution level also determines whether such a hydrophilic characteristic is present or not and depends on the fractal particle agglomerates. For *θ* = 90°, the droplet partially wets the droplet surface and shows a hydrophilic to hydrophobic transition. For the hydrophobic particle surface at *θ* = 120°, the droplet shows its early detachment upon contacting the surface. The corresponding consequences have already been discussed with respect to Fig. 9.

**FIG. 13.**
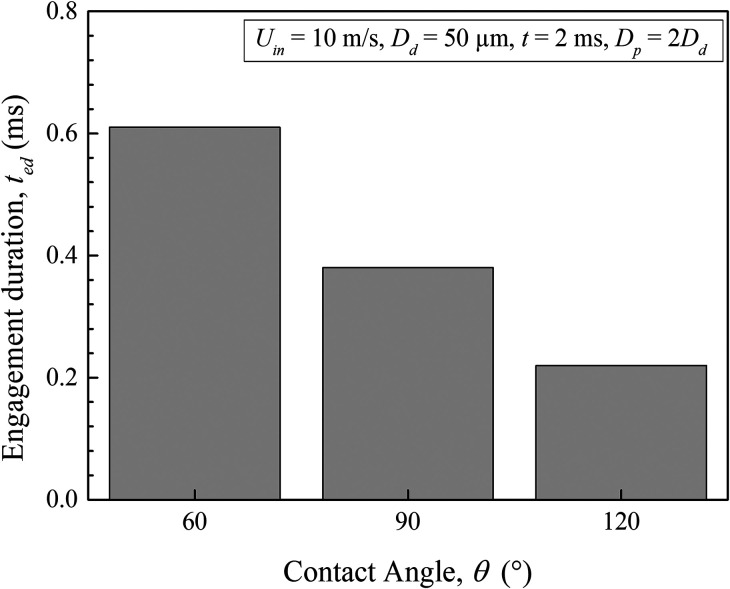
Duration of engagement for a droplet and the air particle with contact angle.

We have seen the droplet spreading characteristics over the particle surface for different contact angles and also the transient evaluation of droplet–particle interaction. Another important question that is very much relevant from the perspective of infected droplet particle transmission through the air is the engagement duration. This determines the corresponding duration of droplet suspension in the air and the effective time of infection propagation from an infected person. To understand this important behavior, we have examined the engagement duration of a droplet with the suspended particles. In Fig. 13, we have plotted the relative dominance of engagement duration for different surface wettability conditions. As obvious, for specific sets of atmospheric conditions, the surface with hydrophilic wettability characteristics exhibits maximum engagement duration of the droplets, whereas for the hydrophobic surface, it is the least. In general, the percentage reduction of engagement duration is 36% when the surface moves toward hydrophobic characteristics from its hydrophilic features. In reality, there might be an additional influence of droplet evaporation, which causes mass transfer from the droplet to the local atmosphere. As a result, the droplet engagement duration may further change. However, the present study only deals with an isothermal model, and hence we have ignored the consequences of droplet–air mass transfer characteristics.

**FIG. 14.**
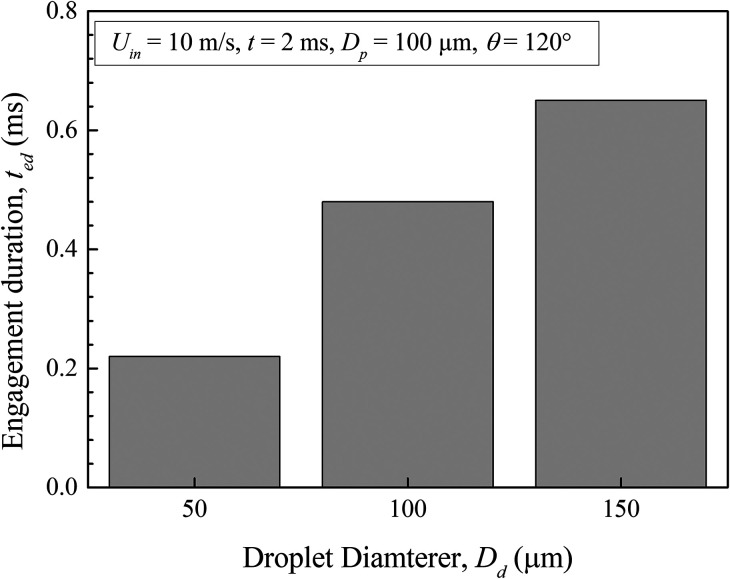
Duration of engagement of a droplet with air particles according to droplet diameter.

During sneezing and coughing, the droplet size significantly varies, and engagement duration also alters. Accordingly, in Fig. 14, we have shown the effect of droplet size on the engagement duration for *U_in_* = 10 m/s. We can see that droplet engagement duration increases with increase in the droplet diameter, and the maximum engagement is found for a droplet diameter of 150 *μ*m considered in this study. As the droplet size increases, the droplet contact area with the particle also increases concomitantly. As a result, the area of the particle surface wetted by the droplet also increases. Depending on the particle size, one may experience complete wetting if the droplet diameter exceeds the particle diameter. Therefore, the time required for a droplet to detach from the particle surface increases, corresponding to enhancement in the engagement duration. In this context, we can conclude that as the droplet emanates from the source, the area near to it contains larger diameter droplets. Therefore, the aerosol particles residing near the source may suffer complete wetting. That triggers the infection propagation, which can be avoided by wearing a mask. Our study also points to the same conclusion as recommended by WHO.^39^ Furthermore, the level of air pollution is affected by the presence of different-sized particulate matters, such as PM10, PM2.5, and PM0.1.^41^ PM2.5 and PM0.1 arise due to emissions from the combustion of fossil fuels,^41^ industrial emission, construction sites, and fires.^42^ Carbonaceous soot is one of the sources of particulate matter, with a variety of hydration properties,^43–45^ such as hydrophobicity and hydrophilicity, due to their chemical composition. Several cities in the world have a high index of PM2.5 and PM10; hence the dynamics of the transport of droplets in the presence of particulate matters significantly change with respect to the cases where particulate matters are not considered in the analysis.

**FIG. 15.**
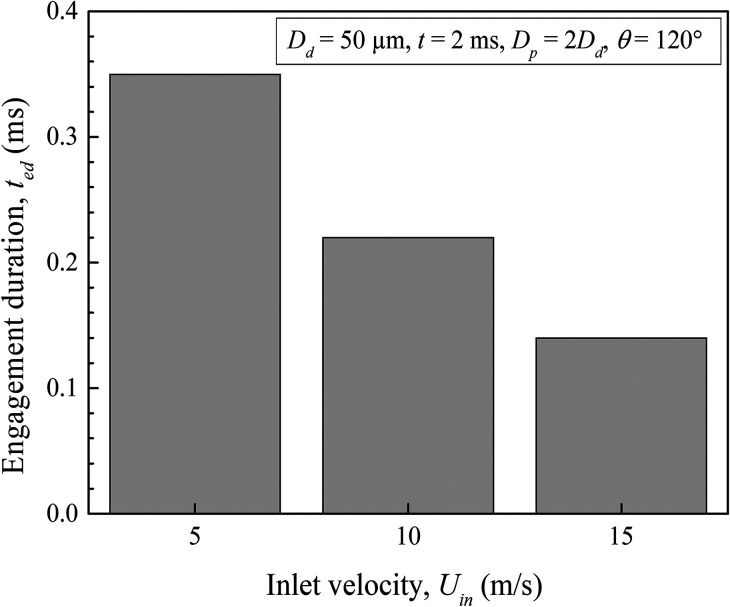
Duration of engagement of a droplet with the air particle at varying inlet velocities.

One may experience fluctuation in the sneezing velocity due to changes in the local atmospheric air velocity. As a result, the droplet's engagement duration varies. To understand such a behavior, we have calculated the droplet engagement duration for various sneezing velocities. In Fig. 15, we have shown the variation in droplet engagement duration for different sneezing velocities. We have noticed that increasing sneezing velocity causes a proportionate reduction in the droplet engagement duration. The maximum droplet engagement is found at a velocity of 5 m/s. We attribute this observation to the enhancement in advection of the droplet compared to its adhesion with the particle, therefore, the corresponding enhancement in engagement duration with reduction in the sneezing velocity. This observation has severe consequences for the transmission of infectious droplets to nearby people, in the case of a healthy athlete or a person during exercise who may exhale droplets with a higher velocity. Hence, it is not recommended to use physical exercise centers in a closed environment such as a gymnasium, indoor games, during the COVID pandemic, especially in a polluted environment. Interestingly, the observation from our simulation experiments again supports the guidelines set by WHO.^46^

It is worth mentioning here that the characteristic of a surface determines the drying time of a respiratory droplet, which requires 25% more drying time than a pure water droplet.^47^ Furthermore, thermocapillary convection inside the droplet, i.e., the Marangoni effect while evaporating on the surface and the air convection outside the droplet, influences the drying time by changing the contact angle.^25^ This drying time increases to hours and even to days, essentially for the survival of a virus.^48^ Thus, decreasing the drying time to reduce the COVID-19 infection by killing the viruses becomes very important. Hence, frequent cleaning of a regularly touched surface is crucial and also it is suggested that heating surfaces at high temperature even for short duration can reduce the chances of the virus survival and infection of COVID-19.^4^ However, we have considered a three-dimensional isothermal model to understand the interaction between ejected droplets and aerosols in the present analysis. We have undertaken such an analysis by observing the current atmospheric pollution levels at different metropolitan cities in the Indian subcontinent. For example, the recent pollution level data released by WHO show that almost all of the global population (99%) breathe air that exceeds WHO guideline limits containing high levels of pollutants, with low- and middle-income countries suffering from the highest exposures. The combined effects of SARS-COVID containing virus droplets and air particulate matters may effectively culminate in the overall virus transmission. However, such a three-dimensional analysis with non-isothermal droplets undergoing evaporation is beyond the scope of this article and we will consider this in our future research investigation.

## IV. CONCLUSIONS

In the present research, we have performed three-dimensional computations to analyze the effect of interactions between respiratory droplets and suspended solid particles in the air on the spreading dynamics of the SARS-CoV-2 infection. Employing a finite volume-based formalism, we have chosen three specific droplet diameters for a particular range of sneezing velocities. The wettability angle during interactions between the droplet and the particles is varied by considering both hydrophilic and hydrophobic interactions. Our simulation results yield the following conclusions:

(1) The level of atmospheric pollution controls the degree of interactions of the droplets during their motion. It is found that the shape of the initially circular droplets takes an elliptical contour by undergoing successive deformations.
(2) The droplets from their source start to disperse at *t* = 2 ms, increasing the lateral distance between them. The overall distribution of the droplets is found to follow a triangular pattern.
(3) The droplets injected with a higher sneezing velocity travel much larger distances, and further enhancement in the sneezing velocity does not show any apparent effect on the lateral dispersion of the droplets. We found a slightly higher dispersion for 10 m/s sneezing velocity.
(4) It is found that based on the local pollution level and the constituent aerosol particles with their wettability characteristics, infectious clouds may propagate a different distance, which eventually affects the social distancing norms for high to moderately polluted areas.
(5) The aerosols with hydrophobic wettability are found to be more prone to the propagation of this disease since it causes complete wetting by the droplets sneezed from the source. A partial wetting of the particles for *θ* = 90° and an early detachment of the droplet from the particle surface at *θ* = 120° are also found.
(6) The droplet engagement duration increases with increase in its diameter, and the maximum engagement duration is noticed for 150 *μ*m diameter droplets.
(7) An enhancement in the sneezing velocity causes a proportionate reduction in the droplet engagement duration, and the maximum droplet engagement is found for a velocity of 5 m/s.

## Data Availability

The data that support the findings of this study are available from the corresponding authors upon reasonable request.
